# Healing of large endodontic lesions using a new combination of triple antibiotics: A case report

**DOI:** 10.1002/ccr3.6879

**Published:** 2023-01-23

**Authors:** Saeed Asgary, Ardavan Parhizkar

**Affiliations:** ^1^ Iranian Centre for Endodontic Research, Research Institute for Dental Sciences Shahid Beheshti University of Medical Sciences Tehran Iran

**Keywords:** antibiotics, endodontics, large lesions, treatment outcome

## Abstract

In the case report presented, three cases of large endodontic lesions, which were the consequences of endodontic treatment failure, were studied. In all cases, a novel combination of triple antibiotics was used to successfully manage and heal the lesions, showing the promising potential of the antibiotic combination in resolving pathosis.

## Abstract 2

The recently introduced combination of triple antibiotics (penicillin G, metronidazole, and ciprofloxacin; PMC) seems to be an effective medication against intracanal microbiota and appears to be able to assist in the preparing a matrix for the healing of endodontic lesions, making PMC a promising medicament against the dental root canal originated diseases.

## 1 INTRODUCTION

Endodontic treatment is principally aimed to ideally eliminate, or remove the maximum number of, microorganisms from the root canal system (RCS), create an insecure environment for the further growth and existence of the remaining bacteria, formulate a setting for possible tissue healing, and prepare a matrix for future ministrations, that is, tissue regeneration and revascularization in regenerative endodontics, and canal obturation and coronal restoration in conventional treatment methods.^1^ Achievement of proper apical and coronal seals to minimize and/or ideally halt the re‐entry of microorganisms into RCS serve the same objectives.^2^ In fact, if microorganisms manage to gain/regain access to the pulp chamber or radicular space, and contaminate/recontaminate the corresponding regions, they are able to cause primary/secondary infections resulting in endodontic pathosis.^3^ Chemomechanical debridement is considered the very first step in order to remove microorganisms from RCS; however, studies have shown that microorganisms can be left behind after chemomechanical preparation and inappropriate root canal treatment, especially in distant areas or places difficult, if not impossible, to reach, that is, fins, cul‐de‐sacs, accessory canals, and isthmuses.^4^, ^5^ Consequently, the application of intracanal medication has been deliberated over the years, using various types of drugs, including antibiotics. In numerous studies, calcium hydroxide (CH) has been widely used as an intracanal medicament to combat regional microbiota.^6^, ^7^ However, it has been recently shown that CH is not as effective as speculated against intracanal microorganisms and cannot be addressed as a strong antibacterial agent despite having an alkaline pH.^8^ Therefore, different studies have investigated new drugs or combination of drugs with more efficient antimicrobial activity as appropriate replacements for CH, for example, antibiotics, to be employed in endodontic treatments. Triple antibiotics paste (TAP), a combination of ciprofloxacin, metronidazole, and minocycline, has been discussed in various investigations for use in regenerative endodontic protocols and disinfection of RCS. Despite acceptable degrees of success in achieving the aforementioned purposes, TAP has shown drawbacks, that is, discoloration of tooth structure and changes in the physical/mechanical properties of radicular dentine, which seem to be in relation with the existence of minocycline in the paste.^1^ As a result, modifications of TAP (mTAP), with cefaclor, amoxyclav, clindamycin, and amoxicillin as possible replacements for minocycline, have been studied; nevertheless, related research has reported degrees of tooth discoloration immediately or after a time period of using mTAP.^9^, ^10^ Some other studies have removed minocycline from the combination without any replacements for the drug; however, the double antibiotic paste comprising ciprofloxacin and metronidazole lack the antibacterial activity related to minocycline.^11^ In a recent study, a novel combination of triple antibiotics was introduced (penicillin G, metronidazole, and ciprofloxacin; PMC), which exhibited a wide range of antibacterial activity with penicillin G as one of its components affecting *Enterococcus faecalis*, a dominant microorganism found in endodontic failure. Moreover, the combination revealed acceptable antibiotic activity against other prevailing intracanal microbiota.^12^

The aim of the current case study was to report the effectiveness of long‐term application of PMC in the healing of large periapical lesions through the disinfection of RCS.

### 1.1 Case 1

A 34‐year‐old male patient was referred for the endodontic retreatment of his maxillary right first molar. The tooth had been endodontically treated ~2 years before the first treatment visit; nonetheless, the patient expressed severe pain and discomfort due to an acute abscess on tooth #3.

**FIGURE 1:**
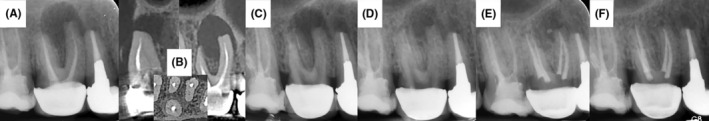
Periapical radiographs and cone‐beam computed tomographic (CBCT) images of case #1: (A) Initial periapical radiography, showing a large endodontic lesion, severe bone destruction, and poor obturation in mesial root. (B) CBCT images in the coronal, axial, and sagittal plans, indicating a large periradicular lesion around the mesial root resorbing the buccal plate, missing the second mesiobuccal canal, and signs of external inflammatory root resorption [EIRR]. (C) Removal of previous endodontic filling materials, chemomechanical preparation of root canals, application of intracanal medication using penicillin G, metronidazole, ciprofloxacin [PMC], and temporization of coronal cavity. (D) 3‐month recall, demonstrating relative resolution of large periradicular lesion around the mesiobuccal root. (E) Obturation of root canals and restoration of coronal cavity with resin‐based dental composite restorative material. (F) 26‐month recall, revealing perfect bone healing, thorough disappearance of periapical lesion, and complete arrest of EIRR.

Medical and dental histories were taken in the first appointment. The clinical intraoral examination showed an intraoral localized abscess on maxillary right molars; however, neither pathological mobility or periodontal pocket nor active pus drainage were noticed. Radiographic evaluation using periapical radiography disclosed a large periradicular radiolucency and severe bone destruction. Additionally, the closer observation of previous endodontic treatment indicated underfilling in the mesiobuccal root (Figure 1A). Cone‐beam computed tomography (CBCT) showed missed second mesiobuccal canal (MB2), poor obturation, large bone destruction, and signs of the external inflammatory resorption of mesial root (Figure 1B). The tooth was diagnosed with acute apical abscess; therefore, the proposed treatment options were thoroughly explained to the patient, comprising: (i) simple extraction of tooth #3 with/without replacement using dental implants or fixed/removable dental prosthesis, (ii) nonsurgical endodontic retreatment (NSER) of tooth #3, and (iii) surgical interventions, that is, apicoectomy or intentional replantation. The patient insisted on retaining the tooth with the most possible conservative approach; consequently, NSER of tooth #3 was selected. Next, the informed consent was obtained from the patient.

In the first treatment session, an appropriate access cavity was prepared without the removal and/or dislocation of the already‐placed single crown on tooth #3. Then, the canal orifices were investigated, located, and penetrated, including the missed MB2 canal. Using chloroform, endodontic filling materials were completely removed and root canals were chemomechanically prepared with copious amounts of 5.25% sodium hypochlorite (NaOCl). After irrigation with normal saline solution, PMC (penicillin G, metronidazole, and ciprofloxacin with the ratio of 1:1:1) was prepared and transferred into the RCS with the succeeding temporization of coronal cavity (Figure 1C).^13^ A week later, the abscess completely subsided and the patient reported no pain on percussion/mastication, and in 3 months, radiographic evaluation revealed the relative resolution of large periradicular radiolucency (Figure 1D). Subsequently, the RCS was cleaned with copious amounts of normal saline, obturated with gutta‐percha/sealer using lateral condensation technique, and the coronal cavity was restored with resin‐based dental composite restorative material (Figure 1E), and the patient was dismissed for regular follow‐ups. In 26‐month recall, the patient reported no sign of discomfort, and the tooth was functional and symptom‐free. Radiographic examination showed complete healing of the large endodontic lesion, normal bone architecture, and re‐establishment of periodontal ligament (PDL; Figure 1F).

### 1.2 Case 2

A 33‐year‐old female patient attended our endodontic center due to severe discomfort and localized abscess in her right mandibular region after performing endodontic treatment on her tooth #30 1 month before the first treatment session. Once detailed medical and dental histories were obtained, clinical examination was conducted and revealed a defective coronal filling on tooth #30, localized gingival abscess adjacent to the bifurcation area and painful response to the percussion test.

**FIGURE 2:**
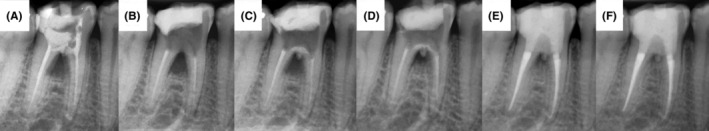
Periapical radiographs of case #2: (A) Initial diagnostic periapical radiography, exhibiting defected coronal restoration, inappropriate previous endodontic treatment, and negotiated bifurcation with large regional radiolucency. (B) Removal of compromised coronal restoration materials, cleaning the pulp chamber, detection of bifurcation involvement, and employment of the novel combination of triple antibiotics [PMC] and temporization of coronal cavity. (C) Repair of furcation perforation using calcium‐enriched mixture cement after the abscess has totally been subsided and re‐employment of the PMC. (D) 3‐month recall; revelation of the relative resolution of bifurcation radiolucency. (E) 4‐month recall; mere disappearance of bifurcation radiolucency, chemomechanical preparation/obturation of root canals with gutta‐perch/sealer and restoration of coronal cavity with resin‐based dental composite restorative material. (F) 10‐month follow‐up; expressing complete resolution of bifurcation lesion, and regional bone healing.

In diagnostic radiography evaluation, poor coronal/root canal filling and bifurcation radiolucency were seen; the furcation seemed to have been negotiated and perforated during the previous treatment (Figure 2A). The tooth was diagnosed with symptomatic apical periodontitis with furcation abscess, and thus, possible treatment options were methodically explained to the patient, consisting of (i) simple extraction of tooth #30 with/without replacement using dental implants or fixed/removable dental prosthesis, (ii) NSER of tooth #30. The patient vowed for the most conservative approach so as to preserve the dental structure in the oral cavity; as a result, the patient agreed on the repair of bifurcation perforation accompanied by the NSER of tooth #30. Succeeding, informed consent was taken from the patient.

The compromised coronal restoration was completely removed, the furcation perforation was instantly located, PMC was prepared (in accordance with the protocol mentioned in case 1) and was transferred to the access cavity, the coronal cavity was finally temporized, and patient was dismissed for 2 weeks (Figure 2B). In 10‐day recall, the abscess totally subsided and the patient reported no pain/discomfort on chewing; thereafter, old PMC was removed, perforated furcation was repaired/sealed with a calcium silicate‐based bioactive [i.e., calcium‐enriched mixture (CEM)] cement, fresh PMC was re‐employed into RCS, and the tooth was temporized (Figure 2C). Three months later, radiographic examination demonstrated a relative resolution of the furcation lesion (Figure 2D). In 4‐month recall, and following clinical/radiographic evaluations showing the progressive resolution of the lesion, coronal dressing was taken out, the old PMC was removed from the access cavity, and the RCS was emptied from the endodontic filling materials using chloroform. Following chemomechanical preparation, RCS was filled/sealed with gutta‐percha/sealer using lateral condensation technique. Consecutively, the coronal cavity was restored with resin‐based dental composite restorative material (Figure 2E). In 10‐month recall, the patient reported no clinical symptoms/discomfort and radiographic evaluation showed favorable outcomes, that is, the complete disappearance of furcation lesion and perfectly new regional bone formation (Figure 2F).

### 1.3 Case 3

**FIGURE 3:**
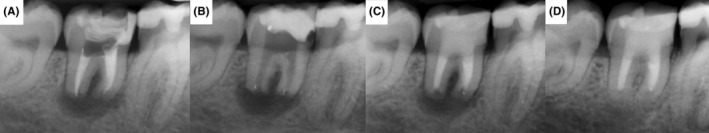
Periapical radiographs of case #3: (A) Preliminary diagnostic periapical radiography; a large periapical lesion and shortened roots caused by external inflammatory root resorption [EIRR] are evident. (B) Previous restorative/endodontic filling materials were removed, PMC was employed and tooth #31 was temporized. (C) 2‐week recall; after the clinical sign/symptoms has totally been subsided, chemomechanical preparation was performed, root canals were filled with a calcium silicate‐based biomaterial, that is, CEM cement, and coronal cavity was restored using a resin‐based dental composite restorative material. (D) 30‐month recall; perfect healing of the periradicular lesion/bone healing and thorough arrest of EIRR are clearly observed.

A 36‐year‐old female patient was referred with a chief complaint of highly sensitive and extremely tender mandibular right second molar with a fragmented coronal restoration. The patient complained about severe discomfort and harsh pain during mastication and a localized small‐sized swelling around tooth #31. In initial periapical radiography, it was shown that the tooth had been previously treated endodontically; however, a large periradicular lesion wrapped around both roots and external inflammatory root resorption (EIRR) had shortened the roots (Figure 3A).

Medical and dental histories were taken in detail, and clinical/paraclinical examinations were conducted. In the intraoral examination, tooth #31 responded painfully to the percussion test and small‐sized confined swelling was discovered adjacent to the involved tooth; however, no active pus secretion was seen. The previous tooth‐crown restoration had been dislodged, carious lesions were observed on the coronal cavity, and grade II mobility was revealed. The tooth was diagnosed with symptomatic apical periodontitis with EIRR; and thus, the treatment options were advised, comprising (i) simple extraction of tooth #30 with/without replacement with dental implants or fixed/removable dental prosthesis, (ii) intentional replantation, and (iii) NSER of tooth #31. The patient wished to preserve her own tooth and demanded the most conservative treatment option. Consequently, NSER of tooth #31 was thoroughly described to the patient, and following the agreement on the chosen ministration, her informed consent was obtained.

Following the removal of previous poor restorative materials and caries, the root canals were accessed, and the used endodontic filling materials were removed. Afterward, PMC was prepared with the same protocol in the previous cases and employed into the RCS. The tooth was temporized, and the patient was dismissed for 2 weeks (Figure 3B). In the next session, the patient reported no complaints regarding pain on the treated tooth, and intraoral clinical examination showed no discomfort on percussion with swelling almost being disappeared from the region. Consequently, the coronal dressing and PMC were removed, and following irrigation/drying out, canals were chemomechanically prepared and then obturated using a calcium silicate‐based biomaterial, that is, CEM cement, and the coronal cavity was filled with resin‐based dental composite restorative material (Figure 3C). After 30 months, the tooth was symptom‐free and intraoral clinical examination demonstrated a functional tooth. In addition, radiographic evaluation revealed perfect healing of the lesion and thorough arrest of EIRR (Figure 3D).

## 2 DISCUSSION

In the presented case study, a new combination of triple antibiotics, that is, PMC, was employed as the intracanal medicament to combat root canal microbiota, encourage bone healing, re‐establish the PDL, and help arrest EIRR. The novel PMC introduces penicillin G as a replacement for minocycline; an antibacterial agent formulated in TAP. Various studies have shown drawbacks for TAP, for example, tooth discoloration, bacterial resistance, and unwelcomed changes in the mechanical properties of dentine.^14^, ^15^ Additionally, minocycline does not have desirable bactericidal/bacteriostatic effect on *Enterococcus (E) faecalis*, a dominant microorganism which can escape antimicrobial means, survive in the involved area, and play an important role in endodontic failure.^16^, ^17^, ^18^ Furthermore, TAP is not capable of acting against *Candida (C) albicans* biofilm and is considered a harmful combination to stem cells.^19^ Nonetheless, penicillin G is a medicament which can affect and remove *E. faecalis*, and act as a bactericidal agent against other gram‐positive intracanal microorganisms.^20^, ^21^ In addition, penicillin G has a white appearance, and as a consequence, it could be speculated that its combination with other medicine having white appearance, that is, ciprofloxacin and metronidazole, may have no discoloring potential. However, well‐designed in vitro and in vivo studies are necessary to evaluate the effect of PMC on tooth discoloration. Besides, the novel combination in the newly formulated triple antibiotics lacks minocycline and should not theoretically deliver the comparable changes in the mechanical properties of dentine as was observed in the application of TAP. Nevertheless, the aforementioned superiority should be carefully experimented.

Apical periodontitis, for example, symptomatic apical periodontitis and apical abscess, is caused by microorganisms and their by‐product which have invaded the periapical/radicular tissues following their departure from RCS.^22^ Various bacteria can be found in the above‐mentioned lesions, for example, *E. faecalis*, *Porphyromonas endodontalis* , and *Streptococcus* species, which seem to be in comparable virulence and dominant microorganisms in the creation of the endodontic lesions.^23^ *Enterococcus faecalis* is a gram‐positive and commensal microorganism considered an opportunistic bacterium and seems to act as a primary pathogen in endodontic failure.^18^, ^24^ *Porphyromonas endodontalis* is a gram‐negative anaerobic rod‐shaped microorganism and is associated with causing endodontic infections; however, it is susceptible to different antibiotics, for example, penicillin and metronidazole.^25^ *Streptococcus* species are gram‐positive, catalase‐negative non‐spore‐forming cocci, which mostly facultative anaerobes although instances of obligate anaerobe can be explored. *Streptococcus* is sensitive to penicillin; nevertheless, there seem to be strains resistant to penicillin, which are susceptible to multiple antibiotics.^26^ Seemingly, PMC can affect these microorganisms and similar microbiota, resulting in the removal of bacteria and preparation of a matrix for further healing.

The postoperative radiographs in follow‐up sessions showed complete resolution of large endodontic lesions of the teeth treated with PMC. The healing observed in the surrounding bone indicates that PMC seemed to be able to eliminate a wide range of microorganisms from the RCS and act satisfactorily against the harmful microbiota by‐products/toxins. These findings of the current case series are in line with the results reported by other investigations, showing that TAP could help in the disappearance of periapical/periradicular lesions.^27^

In the different courses of performed treatments in the investigated cases, PMC managed to control and regress periapical lesions, and encourage bone healing. Therefore, the removal of the causative microorganisms, as the etiologic factor, plays a significant role in the healing of soft and hard tissues. This finding is in agreement with the results found in other studies expressing that using various medicaments in RCS can contribute to tissue healing and reaching successful results.^28^, ^29^ Moreover, in the studied cases, after the termination of treatments, the teeth clinically remained symptomless and non‐obturated, and radiographically exhibited thorough healing of large endodontic lesions. The obturation of RCS was postponed for the period at which initial healing was evident and clinical symptoms were subsided. The protocol used in this stage of the current study was in line with the research conducted by Shah et al., who showed healing of endodontic lesions without root canal obturation in initial stages.^30^ Nevertheless, other studies have shown the importance of root canal obturation in the achievement of success in root canal therapy.^31^ Consequently, the canals were obturated to achieve the long‐term success.

The cases reported in the current research were trailed for different time periods, that is, 26‐, 10‐, and 30‐month follow‐ups for cases 1, 2, and 3, respectively. These follow‐up periods are similar to that of a study by Kumar et al., who conducted their treatment over a minimum period of 12 months to observe the healing of periapical lesion employing intracanal medication, although the complete healing occurred within 1–2 years.^27^ In another study, Brogni et al. introduced a follow‐up period of 15–24 months to present their findings on the healing of periradicular pathosis using TAP, CH, and chlorhexidine as intracanal medication.^32^ Furthermore, Asgary et al. performed their investigation to present their outcomes on non‐obturated root canals and re‐establishment of PDL over 12 months, whereas Shah et al. chose a period of 6 years to report their findings on the delayed root canal obturation.^33^, ^34^ Seemingly, the longer the follow‐up period(s), the more reliable the results will be. The completeness of follow‐ups/recalls is deliberated as a criterion for the assessment and/or evaluation of successful outcomes in conducted research or studies.^35^

In the first and third cases presented, signs of shortened roots owing to hard tissue resorption, that is, EIRR, was reported, managed, and thus, treated afterward. External Inflammatory root resorption is a disturbing and deteriorating complication where dental hard tissue is chelated and/or removed due to osteoclastic activity, that is, continuous loss of dentine and cementum as a consequence of clastic cell activity.^36^ Various studies claim that bacterial infection, as one of the major predisposing factors, could be the main cause of EIRR and can initiate the undesirable phenomenon.^37^ Therefore, if the microbial activity is limited, halted, and managed, EIRR can be arrested, a desirable outcome evident in the first and third cases. It seems that the novel PMC can affect the active microorganisms, and through the removal of the involved bacteria, could trigger/promote the healing and management of EIRR.

## 3 CONCLUSIONS

The outcomes of the presented case series showed that the application of PMC as the intracanal medication is able to eliminate the active intracanal microorganisms and play an important part in the management and healing of large endodontic lesions. The newly introduced PMC could be a capable and promising intracanal medicament for the treatment of large periapical lesions and other maladies, that is, EIRR. Further experimental and clinical investigations as well as well‐designed randomized controlled trials are nevertheless needed for more evidence‐based evaluations.

## AUTHOR CONTRIBUTIONS

**Saeed Asgary:** Conceptualization; data curation; formal analysis; investigation; methodology; project administration; supervision; validation; visualization; writing – original draft; writing – review and editing. **Ardavan Parhizkar:** Conceptualization; formal analysis; investigation; methodology; project administration; validation; visualization; writing – original draft; writing – review and editing.

## FUNDING INFORMATION

None.

## CONFLICT OF INTEREST

The authors declare that there are no conflicts of interest regarding the publication of this paper.

## CONSENT

Written informed consent was obtained from the patients to publish this report in accordance with the journal's patient consent policy.

## Data Availability

The data used to support the findings of this study are available from the corresponding author upon request.
